# Evaluating the Role of Metastable Surfaces in Mechanochemical Reduction of Molybdenum Oxide

**DOI:** 10.1021/jacsau.4c00758

**Published:** 2024-11-19

**Authors:** Neung-Kyung Yu, Letícia F. Rasteiro, Van Son Nguyen, Kinga M. Gołąbek, Carsten Sievers, Andrew J. Medford

**Affiliations:** School of Chemical & Biomolecular Engineering, Georgia Institute of Technology, Atlanta, Georgia 30332, United States

**Keywords:** mechanochemistry, MoO_3_ reduction, DFT, ball milling

## Abstract

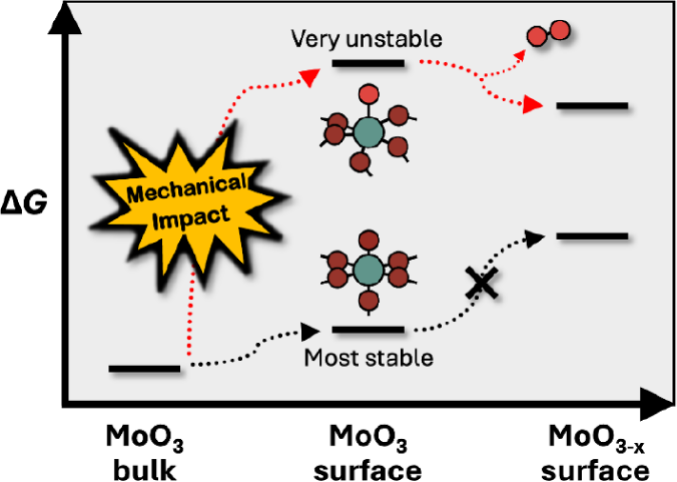

Mechanochemistry and mechanocatalysis are gaining increasing attention as environmentally friendly chemical processes because of their solvent-free nature and scalability. Significant effort has been devoted for studying continuum-scale phenomena in mechanochemistry, such as temperature and pressure gradients, but the atomic-scale mechanisms remain relatively unexplored. In this work, we focus on the mechanochemical reduction of MoO_3_ as a case study. We use experimental techniques to determine the mechanochemical reduction conditions and density functional theory (DFT) simulations to establish an atomistic framework for identifying the metastable surfaces that are most likely to enable this process. Our results show that metastable surfaces can significantly lower or remove thermodynamic barriers for surface reduction and that kinetic energy from milling can facilitate the formation of metastable surfaces that have high surface fracture energies and are not thermally accessible. These findings indicate that metastable surfaces are an important aspect of mechanochemistry along with hot spots and other continuum-scale phenomena.

## Introduction

Chemical reactions generally require energy input to overcome kinetic barriers or achieve thermodynamically favorable conditions unless they are spontaneous. The established fields of thermochemistry, electrochemistry, and photochemistry involve the use of thermal energy, electrical potential, and photons as energetic driving forces for chemical reactions. In contrast, mechanochemistry uses mechanical energy to drive reactions. Although mechanochemistry has a long history, it has recently gained increasing interest due to its potential to enable more sustainable chemical processes and efficient energy conversion, without the use of hazardous solvents.^1,2^ Mechanocatalysis has been demonstrated for various reactions, such as CO oxidation on several materials, including Au, Cr_2_O_3_, and Cu-based catalysts,^3,4^ CO_2_ release from CaCO_3_,^5^ and NH_3_ synthesis on TiN^6,7^ and Fe.^8^

In comparison with many other fields of chemistry, the fundamental mechanisms of mechanochemistry are not well understood. Ball milling, a key technique in mechanochemistry, involves grinding materials in an agitated vessel with milling media.^9^ The process generates mechanical forces, such as friction, shear, and impact, which activate the milled materials and facilitate chemical reactions. Mechanical activation includes the formation of local high-temperature zones, increased surface area through particle fracture, phase transformations, and introduction of structural defects. Despite its promising outlook, comprehensive theoretical understanding of mechanochemistry remains elusive due to the numerous phenomena involved, the transient nature of mechanochemical environments, and the limited availability of experimental methods for in situ or operando observation of mechanochemical transformations.^10,11^

**Figure 1:**
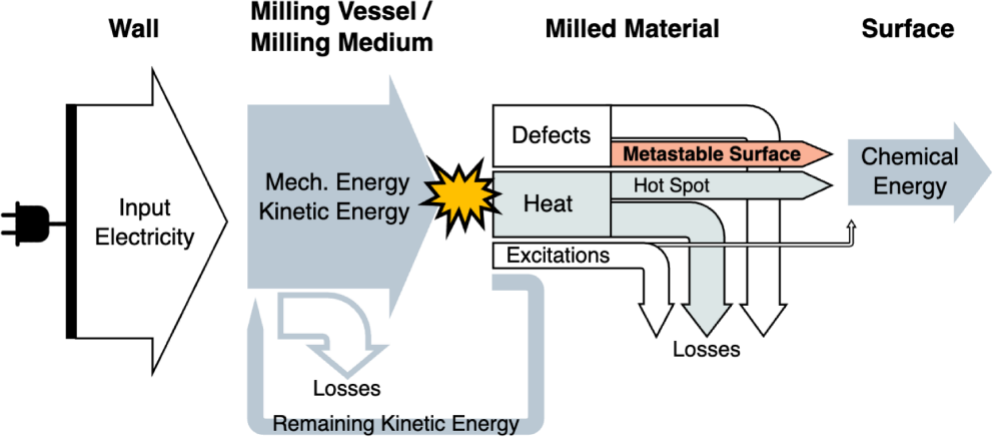
Energy flow diagram during a mechanochemical process.

Mechanochemistry is a complex process that involves many different mechanisms of energy transfer. The relative importance of these mechanisms is not currently well understood, but an illustrative energy flow diagram is depicted in Figure 1. At the macroscale, the input electricity, corresponding to the total energy input, is converted to mechanical energy through electromechanical components such as a motor. This mechanical energy is then transformed to the kinetic energy of the milling vessel and ball. Due to the slight elastic component of the nearly inelastic collision,^12^ a small fraction of initial kinetic energy remains within the milling medium. During the initial fracturing stage of milling, a greater proportion of kinetic energy would be utilized to fracture the milled materials compared to the later stage.^13,14^ In the later deformation stage, the particle sizes are smaller and the rate of size reduction slows significantly. As the energy is further distributed on a smaller scale, it becomes critical for initiating chemical transformations. Specifically, kinetic energy is transferred to the milled materials in the form of mechanical activation, such as heat, defects, and various kinds of excitations.^9^ The portion of the energy corresponding to hot spots and metastable surfaces would be primarily converted to chemical energy by facilitating mechanochemical reactions. The efficiency of energy transfer through the complete mechanochemical process will depend on the optimization of the reactor and the mechanical properties of the mill, catalyst material, and substrate, while the efficiency of the final reaction stage will depend more on the surface chemistry of the catalytic material. Therefore, the rational design of mechanocatalytic materials requires an improved understanding of the fundamental processes that govern the energy transfer between metastable surfaces and chemical reactions.

One of the most studied mechanochemical phenomena is the hot spot, a localized high-temperature region where energy is concentrated via collisions. The hot spot has been proposed as a key factor in facilitating mechanochemical reactions, where the thermal energy helps to overcome activation barriers,^15,16^ although this view is still somewhat controversial.^17,18^ For modeling mechanochemical reactions, many studies have focused on the hot spot theory, primarily using macro- or reactor-scale models based on a continuum approach to capture temperature variations within the system.^19−21^ Another consequence of mechanical impact is structural deformation, which leads to the molecular deformation^18,22^ or the creation of unstable or metastable surfaces^16,23^ that may exhibit enhanced reactivity. Although the formation of metastable surfaces with high surface energy is not thermodynamically favored, milling can lead to particle fracture, exposing unstable surfaces.^23^ This effect offers a plausible explanation for certain mechanochemical phenomena that hot spot theory fails to account for. For example, a study on mechanochemical CO oxidation demonstrated that catalytic activity remained elevated for minutes or hours after milling ceased,^3^ surpassing the relaxation time scale of hot spots. We hypothesize that active sites on metastable surfaces are the main contributors to prolonged catalytic activity after milling has stopped. To further explore this hypothesis, detailed atomistic modeling of these metastable surfaces is necessary. Previous computational atomistic studies of mechanochemistry have mainly focused on the effects of molecular deformation and the forces that act on molecules;^24−29^ however, studies on surface deformation have been limited.^8,30^ One study examined the effect of strain-induced structural deformation and its dynamic relaxation during mechanochemical ammonia synthesis,^8^ but the time scales for relaxation of these strained surfaces were not considered. In molecular dynamics studies of silica and diamond-like materials, strain-induced stress relaxation times are reported to be in picosecond range,^31,32^ which are much faster than typical time scales for chemical reactions.^33−35^ On the contrary, the microsecond time scale of collision^20^ where the ball is in contact with the material is considerably longer than the time scale of strain relaxation or fast chemical reaction, suggesting that detailed dynamic models may be required to assess the mechanisms of reactions that occur during collisions. However, the formation of additional types of metastable surfaces with larger kinetic barriers to relaxation, such as different surface facets, substitutions, or vacancies, can lead to defects that persist well after collisions and may explain the prolonged catalytic activity after milling is stopped.

In this work, we use mechanochemical reduction of MoO_3_ to MoO_2_ as a case study to evaluate the role of metastable surfaces in mechanochemical reactions. Experimental investigations reveal that MoO_3_ is reduced to MoO_2_ when ball-milled in an inert environment, and DFT simulations indicate that the formation of oxygen vacancies on the stable surfaces of MoO_3_ leads to kinetic barriers that are likely insurmountable on the time scale of hot spot formation and dissipation. We use DFT to evaluate the formation energy and thermodynamics of oxygen vacancy formation for various metastable facets and surfaces of MoO_3_, and we show that reduction is feasible even at relatively low temperatures. The findings introduce a framework for identifying the most stable and efficient surfaces for mechanochemical reactions and suggest that metastable surfaces play an important role in mechanochemistry alongside hot spots and other phenomena.

## Methods

### Experiments

#### Mechanochemical Experiments

Molybdenum trioxide (≥99.5%) (MoO_3_) used in these experiments was purchased from Sigma-Aldrich. Before being used, MoO_3_ was subjected to heat treatment at 450^◦^C under air for 2 h to remove any undesired physisorbed molecules. Experiments were conducted in a Retsch MM400 shaker mill. The stainless-steel reactor vessel used had an internal volume of 50 mL and Swagelok-fitted openings on the cylindrical face for gas flow. One grinding ball of 20 mm and 1 g of MoO_3_ were used in the experiments, with the frequency set to 30 Hz based on other tests, and the milling time was varied. All the experiments were conducted under an inert atmosphere, with argon flowing through the vessel at a rate of 60 sccm. For comparison, one sample was prepared using a closed vessel exposed to air atmosphere for 30 min at 30 Hz.

#### Sample Characterization

X-ray diffraction (XRD) of the samples was performed using a Rigaku MiniFlex diffractometer with CuKα1 = 1.540598 Å and CuKα2 = 1.544426 Å. Data were collected in the 2θ range from 10^◦^ to 80^◦^, with a scan step of 0.01^◦^ and a speed of 20.0 ^◦^min^–1^. The crystalline phases of the samples were identified using the Crystallographica Search Match software. Subsequently, the Rietveld refinement method was employed with TOPAS 5 software to quantify these phases and calculate the crystallite sizes. X-ray absorption spectroscopy (XAS) measurements at the molybdenum K-edge were conducted at the ISS (8-ID) beamline of National Synchrotron Light Source II at Brookhaven National Laboratory (BNL). The monochromator used (Si(111)) was calibrated by adjusting the first inflection point to match the K-edge spectrum of a metallic foil standard (20000 eV for molybdenum). Multiple scans were accumulated and later merged to enhance the signal-to-noise ratio. The XANES data were processed using Athena software as well as molybdenum speciation by means of the linear combination (LC) of the spectra. Six merged spectra were used in order to improve the signal-to-noise ratio and the references used for quantitative analyses were MoO_3_, MoO_2_, and metallic molybdenum foil. Details and results of additional characterization, including Raman spectroscopy, particle size analysis from dynamic light scattering, scanning electron microscopy, and X-ray photoelectron spectroscopy (XPS), are provided in the Supporting Information. An XPS survey scan was used to (Figure S9) confirm that there was no iron contamination from the stainless steel.

### Calculations

Density functional theory (DFT) calculations were performed using the Vienna *ab initio* Simulation Package (VASP) version 6.1.2, employing projector-augmented wave (PAW) pseudopotentials.^36−39^ The default pseudopotentials for Mo (4d^5^5s^1^) and O (2s^2^2p^4^) were utilized. The rev-vdW-DF2 functional, which is a generalized gradient approximation (GGA) functional augmented with a nonlocal correlation functional to describe van der Waals (vdW) interactions,^40,41^ was employed. The functional choice was motivated by the need for accurate treatment of vdW interactions due to the layered structure of MoO_3_. A comparison of different possibilities revealed that the rev-vdW-DF2 functional demonstrated strong performance in describing adsorption on the MoO_3_ surface,^42^ as well as in the bulk phase diagram of molybdenum oxides (Figure S1). A kinetic energy cutoff of 520 eV and k-point grids of [round(20/A), round(20/B), 1] were applied (where A and B are, respectively, the x and y dimensions of the supercell and round implies rounding to the nearest integer), and spin polarization was considered with initial guesses of magnetic moments of 1μ_*B*_ for O atoms and 5μ_*B*_ for Mo atoms. The force criterion for structural optimization was set at 0.05 eV/Å. The optimized lattice parameters (a, b, c) for MoO_3_ are 3.93, 14.12, and 3.72 Å, respectively, showing good agreement with the experimental values of 3.96, 13.86, and 3.70 Å.^43^

The Gibbs free energies were obtained by applying thermal corrections only to gas species, assuming ideal gas behavior. The chemical potential of oxygen μ_O_ is computed as
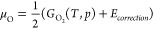
1where , and the thermodynamic quantities are obtained using the thermochemistry.IdealGasThermo class of the Atomic Simulation Environment package,^44^ with vibrational energies calculated using DFT. The *E*_*correction*_ (0.488 eV) is applied to the O_2_ energy to ensure that the formation energy of H_2_O matches the experimental value.^45^

The Gibbs free energy of formation for MoO_3_ surfaces is defined, referenced to MoO_3_ bulk and O_2_ molecules, as
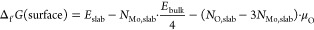
2where  and  are the numbers of Mo and O atoms in a symmetric MoO_3_ slab structure, respectively, and *E*_bulk_ is divided by 4 to represent MoO_3_ bulk energy per Mo atom. This formation energy is computed “per slab”, so it can be interpreted as the energy required to create two metastable surface sites, which corresponds to the top and bottom surfaces, as defined by the boundaries of the supercell. The Gibbs free energy of reaction for O_2_ release is defined as
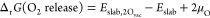
3where  is the energy of a slab with two oxygen vacancies, generated by removing a symmetrically equivalent oxygen atom from both the top and bottom of a symmetric slab. This would be equivalent to the energy required to create two oxygen vacancies that are not in close proximity and is expected to provide a lower bound on the energy required to form two oxygen vacancies in close proximity (as would occur when an O_2_ molecule is released in reality).

## Results and Discussion

### Experimental Mechanochemical Reduction of MoO_3_

MoO_3_ samples before and after milling in an Ar stream were subjected to XRD analysis to assess changes in their crystalline structures after varying milling durations. Figure S3a presents the XRD patterns for the samples along with the standards for MoO_3_ (PDF No. 5-508) and MoO_2_ (PDF No. 76-1807). The data clearly show that all samples exhibited lower peak intensities compared with the initial MoO_3_, suggesting a decrease in crystallinity. This observation is corroborated by the quantification of the crystallite size illustrated in Figure S3b, which demonstrates a progressive reduction in crystallite size with increasing milling time, regardless of the milling atmosphere. The decrease in crystallite size occured along with a decrease in particle size, as measured by dynamic light scattering (Figure S7) and corroborated by scanning electron microscopy (Figure S8).

Rietveld refinement was used to quantify the crystalline phases within the samples, as detailed in Table S1. After 5 min of milling, a small amount of crystalline MoO_2_ was formed (3.4%). With prolonged milling, the proportion of reduced MoO_2_ increased until 60 min. These findings were corroborated by Raman spectroscopy, which revealed characteristic peaks of MoO_2_ after 60 min of milling (Figure S6). To evaluate the impact of atmospheric conditions on the reduction process, a comparative experiment was conducted in air. The sample milled for 30 min at 30 Hz in air showed the formation of 7.3% of a crystalline MoO_2_ phase, while the sample milled under an inert atmosphere exhibited 22.9% MoO_2_. This indicates that mechanochemical reduction occurs even under oxidizing conditions, a result that is not observed in thermoreduction processes.^46^ However, an inert atmosphere is crucial for achieving higher levels of MoO_2_ formation, which we attribute to the reincorporation of oxygen into the lattice under oxidizing conditions. In other words, mechanochemical reduction reaction is reversible and appears to approach an equilibrium between MoO_3_ and MoO_2_ when milled in air, as can be seen in Figures S4 and S5.

**Figure 2:**
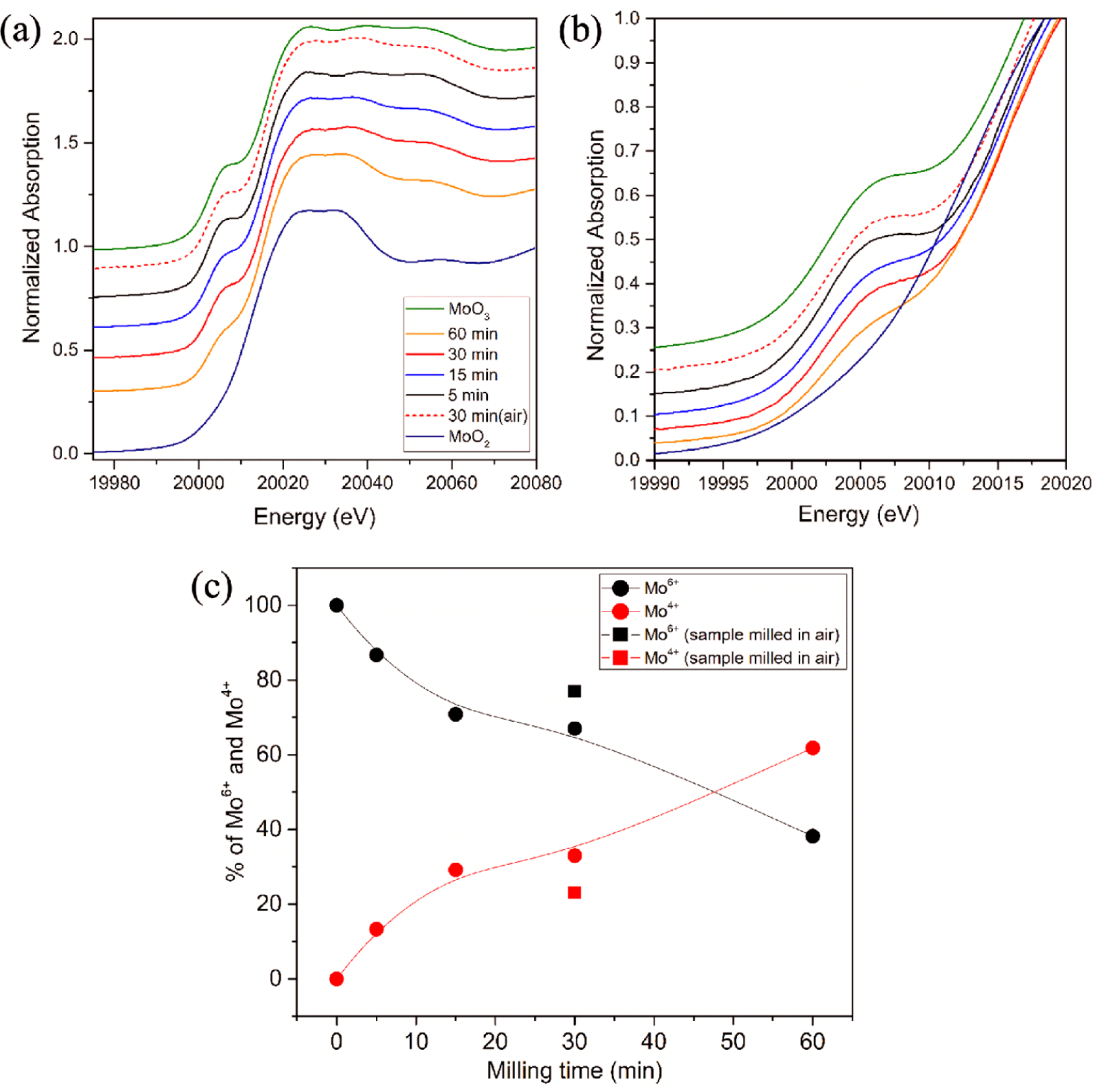
(a) Mo K-edge XANES spectra for the samples and standards (MoO_3_ and MoO_2_). (b) Magnification of the pre-edge region. (c) Quantification of Mo(6+) and Mo(4+) in samples subjected to different milling times at 30 Hz by using a linear combination of XANES spectra.

X-ray absorption spectroscopy was employed to quantify the reduction degree of the entire samples as XRD alone can provide only semiquantitative information about the crystalline phases. According to the literature, milled materials can become highly amorphous, leading to an underestimation of reduction when quantified solely by XRD.^47^Figure 2a presents the Mo K-edge XANES spectra for the samples, while Figure 2b highlights the pre-edge region for easier comparison. The pre-edge shoulder decreased for all of the milled samples, and the K-edge shifted to lower energies, indicating that the different milling times led to varying degrees of sample reduction. These observations were confirmed through linear combinations of the XANES spectra shown in Figure 2c, which revealed that the reduction of Mo(6+) to Mo(4+) reached 13.3% after 5 min of milling and 61.8% after 60 min. These values are higher than those from XRD quantification of the crystalline MoO_2_ phase, suggesting the presence of amorphous reduced Mo species. The rapid decrease of crystallite and particle size in the first 15 min of milling and slower decrease occurring throughout the remainder of milling period (Figures S3b and S7) indicate that more than half of reduction occurs during the later deformation stage of milling. The decreased particle sizes provide more exposed surfaces and defects that likely accelerate the reduction rate. Additionally, the sample milled under an air atmosphere for 30 min reached 23% Mo(4+) compared to 33% of the sample milled for the same time under Ar. These findings underscore the importance of longer milling times and an inert atmosphere for increased Mo reduction.

Without milling, the reduction of MoO_3_ to MoO_2_ begins to be observed after 1–3 h at 723–873 K in inert atmospheres, and requires higher temperatures for a significant reduction.^48,49^ The reduction of MoO_3_ at nominally ambient temperature during milling suggests that mechanical energy enables this reaction by producing hot spots and metastable surfaces.

### Evaluation of Reduction via Stable Surfaces and Hot Spots

It is well established that hot spots can form inside ball mills, with reported temperatures estimated to exceed 1000 K.^16,20^ Given that purely thermal reduction of MoO_3_ can occur at temperatures of 700–900 K,^48,49^ it seems plausible that hot spots could cause this transformation. However, this neglects the time scales required for reduction, which can take minutes to hours under purely thermal conditions.^48,49^ If hot spots are solely responsible for the reduction, then the reduction must occur before the heat from the hot spot dissipates. This dissipation time has been estimated to be ∼400 ms in similar systems^20^ suggesting that additional phenomena beyond the hot spot must be considered to explain mechanochemical reduction.

Mechanochemistry can accelerate the reduction in several ways beyond the generation of thermal gradients. Solid-state diffusion of oxygen can be enhanced by the mechanical mixing of surface, subsurface, and bulk oxygen as new surfaces are formed during milling. The milling process also decreases the particle size due to fracture, which decreases the diffusion length scales for bulk reduction. In addition, milling will produce surface defects that can accelerate the reduction. Ultimately, the reduction process must proceed through a surface; therefore, the energetic barriers for surface reduction provide a starting point for analyzing reduction rates.

**Figure 3:**
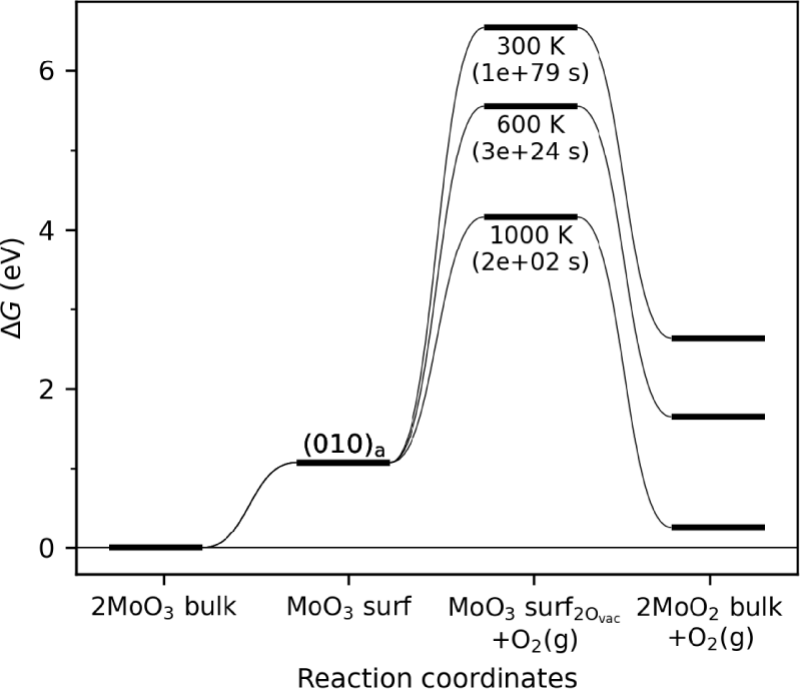
Free energy diagram for mechanochemical MoO_3_ reduction, which includes surface formation and release of the O_2_ on the most stable (010) surface at temperatures of 300, 600, and 1000 K and an O_2_ pressure of 10^–5^ Bar. Thermodynamic corrections were applied only to gas species. Numbers in parentheses represent the inverse of reaction rates.

Atomistic models can provide information about the thermodynamic barrier for the surface reduction. Here, we assume that reduction occurs via the most stable (010) surface facet and evaluate the energetic pathway for reduction at various temperatures. The results in Figure 3 reveal that the formation of two oxygen vacancies in the (010) facet is uphill by ∼3.1 eV even at 1000 K. Using the Eyring equation, and neglecting all additional kinetic barriers, this corresponds to a rate of ∼0.006 s^–1^ (or a time scale of ∼200 s) at 1000 K. Although simplistic, this analysis is expected to provide an overestimate of the rate since it neglects explicit kinetic barriers for vacancy formation and O_2_ release, does not account for repulsive interactions between nearby oxygen vacancies, and assumes peak hot spot temperatures. Even with these approximations, the analysis indicates that the time scales required to overcome the thermodynamic barriers for the reduction of MoO_3_ on the most stable (010) surface are comparable to or longer than the time scales of heat dissipation. Importantly, this analysis neglects the contribution of defect sites, which are expected to have lower barriers to the formation of oxygen vacancies. These results suggest that accounting for defects is critical to establishing an atomic-scale understanding of how the mechanochemical reduction of MoO_3_ occurs, even when high temperatures of hot spots are considered.

### Identification of Metastable Sites Based on Stability and Reactivity

To examine whether metastable surfaces and defects exhibit increased reactivity for O_2_ release, a DFT-based thermodynamic study was conducted. The metastable surfaces were studied by generating symmetric MoO_3_ surface slabs with Miller indices ≤1 and surface area of around 1 nm^2^, considering all possible terminations. The interfaces chosen here likely underrepresent the ruggedness of newly formed cracks, but simulating a much higher indexed surface would be prohibitive in terms of computational resources required. The O_2_ release reaction on the generated surfaces was simulated by removing an O atom from the top and bottom of a slab, and the reaction energy for forming O_2_ from these two O atoms was calculated. Each symmetrically distinct oxygen atom on the surface was treated as a possible defect site, yielding multiple oxygen vacancy formation energies for a surface with the same Miller index and termination. In addition, the (010) and (111) surfaces were subjected to compressive strain (5, 10% in the z direction) and tensile strain (0–25% of compressive strain in the x and y directions, based on Poisson’s ratio) since residual strain is expected in ball-milled materials, and strained surfaces have previously been proposed to be relevant in mechanocatalysis.^8^ However, we did not consider the dynamic relaxation model in their work, as we do not expect that the energy released from strain relaxation would change the thermodynamics. Our approach to considering possible structures during milling is similar to previous work on amorphous materials^50^ in that a wide range of structures are considered to model nonequilibrium states with unclear geometries.

**Figure 4:**
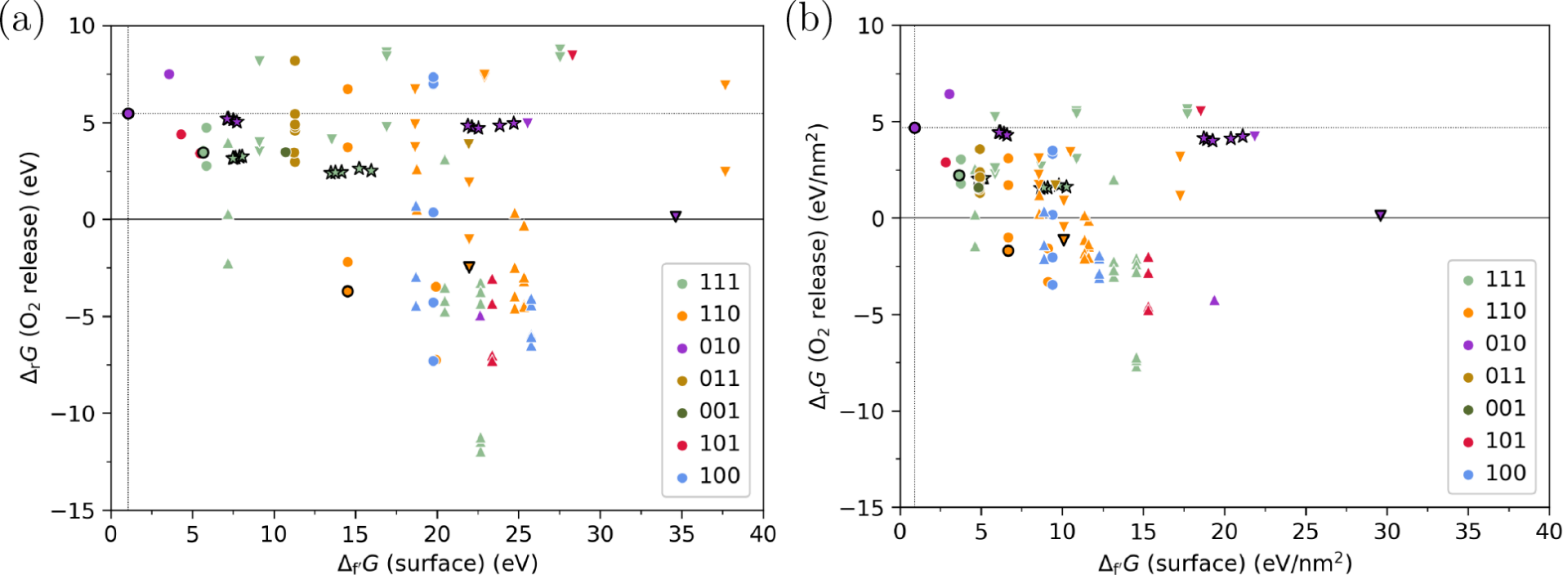
Pareto plot of O_2_ release energy ((O_2_ release)) versus MoO_3_ surface formation energy ((surface)), in eV (a) or eV nm^–2^ (b), both at a temperature of 300 K and O_2_ pressure of 1×10^–5^ bar. Thermodynamic corrections were applied only to gas species. Stoichiometric surfaces are shown as circles, whereas suboxide and superoxide surfaces are shown as downward and upright triangles, respectively. Points with black edges represent the Pareto frontiers considered in the reaction diagrams in Figure 5, with strained surfaces shown as stars.

Based on the Gibbs free energy of MoO_3_ surface formation and the O_2_ release reaction, the difficulty of forming different surfaces and their reactivity were evaluated using a Pareto plot in Figure 4. The plot is based on the free energies of O_2_ release at 300 K to help deconvolute the effects of metastable sites and hot spots, but the qualitative trends are similar at higher temperatures (see Figure S2). The figure shows both the surface formation energy (Figure 4a) and the standard surface energy (Figure 4b). The surface formation energy is equal to the surface energy multiplied by the sum of the areas (top and bottom) and represents the amount of energy required to form two reactive sites. Points with the same (surface) but different (O_2_ release) represent the O_2_ release of symmetrically distinct oxygen atoms on the same surface. Suboxide and superoxide surfaces represent the surfaces having fewer or more oxygen atoms than stoichiometric surfaces. The DFT results correctly identify the (010) facet as the most stable surface,^51^ which is set as a reference point, since we are interested in the additional energy needed to form less stable surfaces and the increased gain of chemical energy through the facilitated reactions. Despite a limited range of surfaces with Miller indices ≤1, the plot demonstrates a wide range of (O_2_ release) and (surface), indicating that a sufficiently diverse set of surfaces is represented. Other higher-index surfaces and amorphous surfaces are likely to participate in the reaction during milling. However, the focus of this study is to provide an initial insight into how the diverse surface configurations formed via mechanical activation can influence mechanochemical O_2_ release. The wide range of (O_2_ release) and (surface) for the sites we have investigated indicate that they are representative of the more complex surfaces that may form, and the conceptual trade-off between these quantities will also govern higher index and amorphous sites.^50^

Analysis of Figure 4 reveals that unstable surfaces are not necessarily reactive to the release of the O_2_ release. Unstable defect sites are sometimes more difficult to reduce than the most stable (010) surface, and strained surfaces are less stable but have similar O_2_ release energies. However, there is a discernible trend that reactivity increases as surfaces become more unstable—increasing the surface formation energy is a necessary but not sufficient condition to facilitate reduction. The Pareto frontier represents surfaces with a higher “mechanochemical efficiency”, meaning that the energy released by the chemical reactions compensates for the energy required to create the surfaces. Notably, this efficiency is not a practical efficiency measure because the additional chemical energy of highly exergonic O_2_ release reactions will be released as heat. Thus, the most relevant sites are those that can easily form (low  (surface)) and spontaneously release O_2_ ((O_2_ release) ≤ 0). Moreover, superoxide surfaces (upright triangles) are not relevant for MoO_3_ reduction, since they are more oxidized than MoO_3_, but these surfaces are included since they may be relevant for other reactions where active oxygen plays a role. There are also numerous stoichiometric and suboxide surfaces that have exergonic O_2_ release, all of which may be relevant for MoO_3_ reduction. We select a subset of four examples for more detailed analysis: one stoichiometric defect that is easier to reduce than the stable (010) surface but still has an endergonic O_2_ release energy, the most stable stoichiometric defect with a strongly exergonic O_2_ release energy, the most stable partially reduced defect with a strongly exergonic O_2_ release energy, and the most unstable partially reduced defect with a slightly endergonic O_2_ release energy.

### Evaluation of Reduction via Metastable Surface Sites

**Figure 5:**
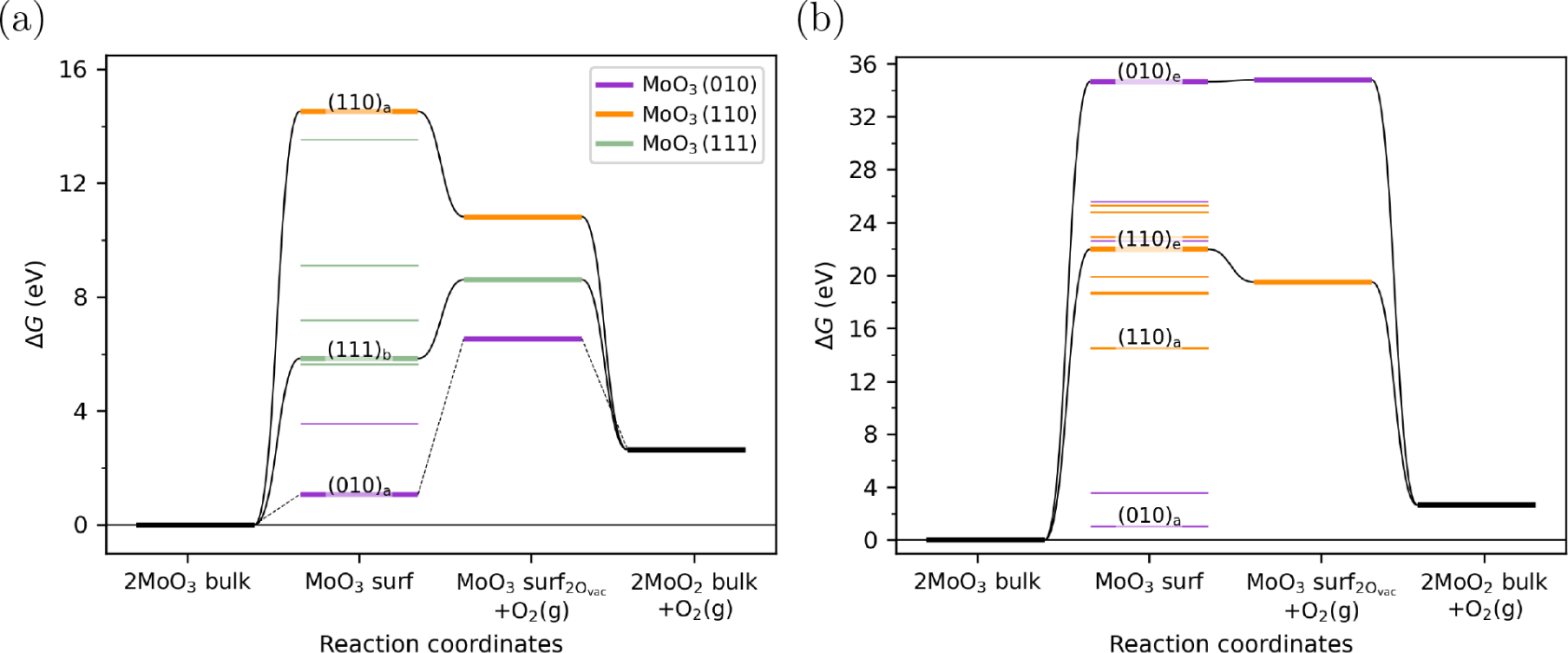
Free energy diagram for mechanochemical MoO_3_ reduction, which includes (metastable) surface formation and O_2_ release on the stoichiometric (a) and suboxide surfaces (b) at a temperature of 300 K and O_2_ pressure of 1 × 10^–5^ bar. Thermodynamic corrections were applied only to gas species.

The Gibbs free energy change during the mechanochemical MoO_3_ reduction at 300 K is presented in Figure 5. The process involves the formation of an MoO_3_ surface from bulk MoO_3_, followed by O_2_ release from the surface, and reduction to bulk MoO_2_. First, we considered surfaces with the same stoichiometry as that of bulk MoO_3_ to study the initial stage of reduction (Figure 5a). The formation of the most stable (010)_a_ surface requires the least energy; however, the subsequent O_2_ release reaction is highly endergonic ( eV when referenced to the created surface). Compared to (010)_a_ surface, creating the (111)_b_ and (110)_a_ surfaces requires considerably more energy, yet O_2_ release is significantly less endergonic or even exergonic ( of 2.8 eV and −3.7 eV on (111)_b_ and (110)_a_, respectively). Comparison with Figure 3 and the corresponding analysis suggests that these surfaces will reduce much more rapidly, particularly if higher temperatures of hot spots are considered. For example, the time scale of reduction for the (111)_b_ surface at 1000 K following the assumptions of Figure 3 is 4 × 10^–12^ s, which is considerably faster than the time scale of hotspot decay (∼400 ms). Meanwhile, the time scale of exergonic reactions based on these simple assumptions would be ∼1 × 10^–13^ s, or effectively instantaneous, even at 300 K. In reality, other processes would become rate-limiting, but the analysis suggests that these metastable sites and defects can be rapidly reduced even in the absence of elevated temperatures. Additionally, the pressure of the O_2_ also affects the energetics. For example, when O_2_ pressure increases to 0.21 bar (as in air)  increases by 0.26 eV (at 300 K) or 0.86 eV (at 1000 K), which helps explain the lower reduction degree of the sample milled in air.

One remarkable observation from Figure 5 is the energy scale, which reaches extreme values of ∼10–30 eV (or similar values in eV nm^–2^, see Figure 4b) for the formation of metastable defects with exergonic O_2_ release. These energy barriers are utterly insurmountable in thermal catalysis—even at 1000 K, the Eyring equation predicts a time scale of ∼1 × 10^37^ s (longer than the age of the universe) to overcome a barrier of 10 eV. However, surface fracture energies in milling can far exceed 100 eV nm^–2^,^52−54^ suggesting that the formation energy for the (110)_a_ surface ( eV for two O vacancies with surface energies of 6.7 eV nm^–2^), is surmountable. As shown in Figure 5b even the most unstable suboxide surfaces, such as (010)_e_, have even higher formation energies ( eV or 29.6 eV nm^–2^), but even these are surmountable with surface fracture energies as a driving force. This stark difference in accessible energy scales highlights a fundamental advantage of mechanochemistry and mechanocatalysis, where high-energy states can be generated even under relatively mild nominal conditions.

## Conclusion

The nature of metastable surfaces and active sites in mechanochemistry is not clear, although it is commonly accepted that a high mechanical energy can induce defects with enhanced activities. Our study has partly clarified this concept through the experimental demonstration of mechanochemical MoO_3_ reduction and atomistic modeling of metastable surfaces with various facets and terminations. We evaluated the trade-off between formation and reduction energy across various surfaces and showed that the reduction becomes spontaneous on some reactive metastable surfaces. We also argue that the large magnitude of surface fracture energies provides a route to generate high-energy metastable surfaces and sites that are not accessible in other types of catalysis. While our current exploration of metastable surfaces is not exhaustive, it introduces a novel and general approach for studying mechanochemistry. Future research should provide a more comprehensive consideration of these surfaces and incorporate kinetic aspects to deepen our understanding of the mechanisms at play.

## Supporting Information Available

Calculated MoO_*x*_ bulk phase diagrams; Pareto plot at a temperature of 1000 K; XRD patterns, crystalline phases, and crystallite sizes of the MoO_3_ samples milled in Ar or O_2_; XANES spectra and quantification of Mo(6+); Raman spectra; particle size measurement by DLS; and SEM images (PDF)
