# Supplementary material for: Evaluating the Role of Metastable Surfaces in Mechanochemical Reduction of Molybdenum Oxide
Source: JACS Au. 2024 Nov 19;5(1):82–90. doi: 10.1021/jacsau.4c00758 (PMC11775685; doi:10.1021/jacsau.4c00758)
Supplement: Supplementary file 1 — au4c00758_si_001.pdf [file au4c00758_si_001.pdf]

Evaluating the role of metastable surfaces in mechanochemical reduction of molybdenum oxide

Neung-Kyung Yu, Letícia F. Rasteiro, Van Son Nguyen, Kinga M. Gołąbek, Carsten Sievers,* and Andrew J. Medford*

School of Chemical & Biomolecular Engineering, Georgia Institute of Technology, Atlanta, Georgia 30332, USA

E-mail: carsten.sievers@chbe.gatech.edu; ajm@gatech.edu

Calculated Bulk Phase Diagrams of MoO_x

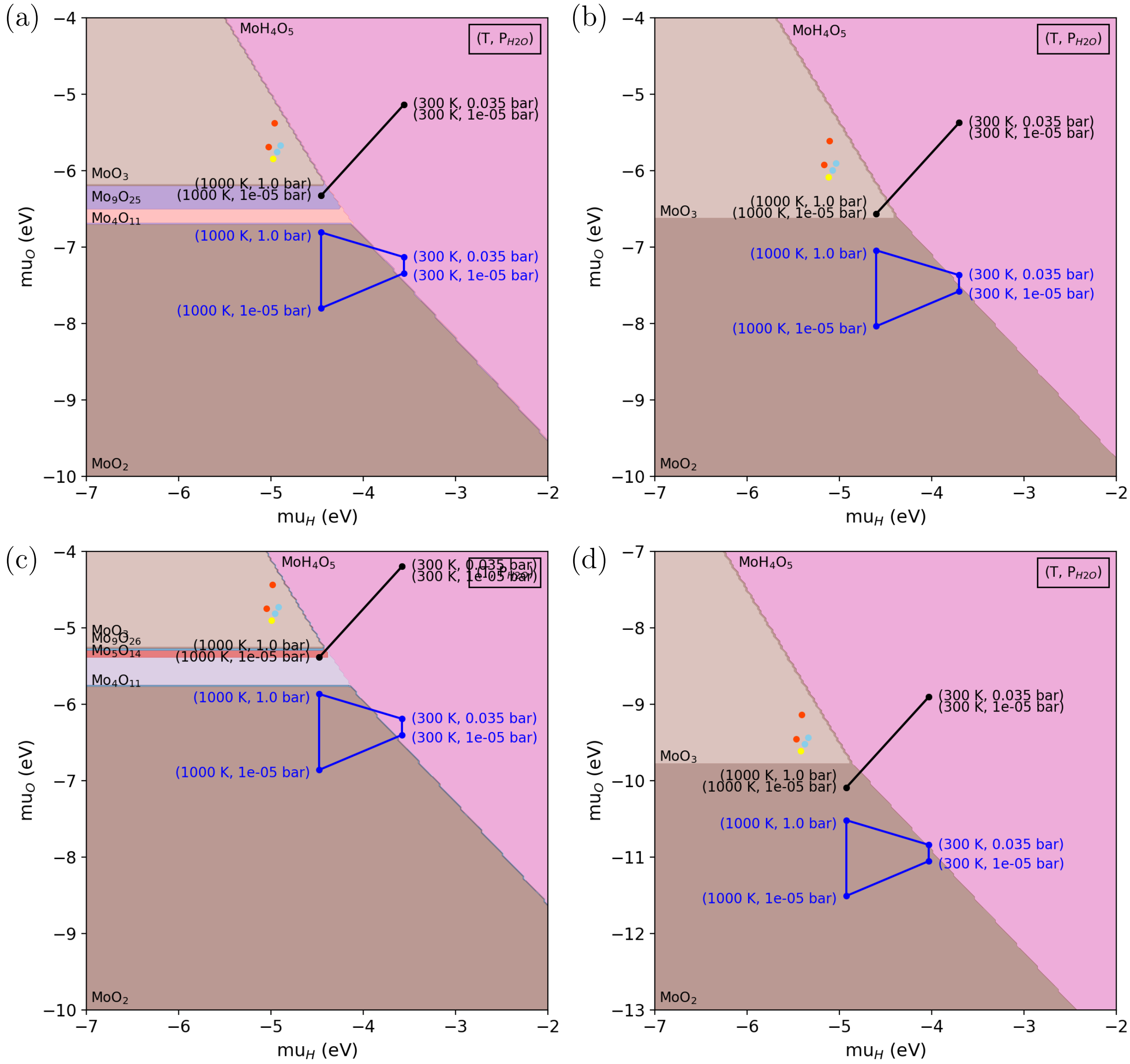

Figure S1: MoO_3 bulk phase diagram calculated using PBE (a), RPBE (b), rev-vdW-DF2 (c), and HSE06 (d) functionals, across ranges of chemical potentials of hydrogen and oxygen. Black and blue points represent the chemical potentials corresponding to the temperatures and H_2O pressures indicated beside them. Red points represent the chemical potentials corresponding to experimental MoO_2 oxidation conditions using O_2 gas or air.[1,2] Sky blue and yellow points represent the chemical potentials corresponding to experimental MoO_3 reduction conditions using H_2 or N_2 gas.[3,4]

Pareto Plot at 1000 K

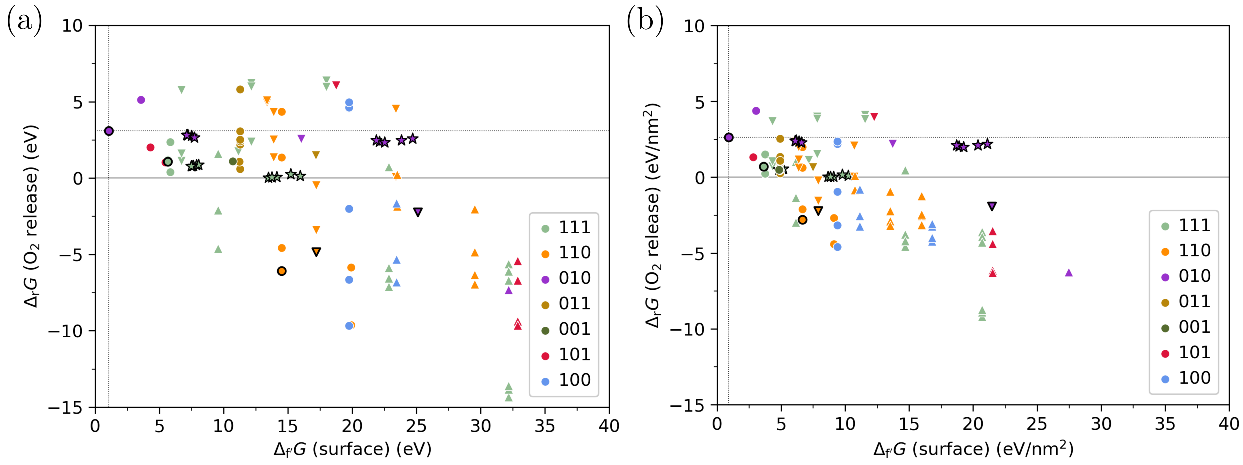

Figure S2: Pareto plot of O_2 release energy ($\Delta_r G(O_2$ release$)$) versus MoO_3 surface formation energy ($\Delta_{f'} G$(surface)), both in eV (a) or eV nm^{-2} (b), at a temperature of 1000 K and O_2 pressure of 1e−5 bar. Thermodynamic corrections were applied only to gas species. Stoichiometric surfaces are shown as circles, whereas sub-oxide and super-oxide surfaces are shown as downward and upright triangles, respectively. Points with black edges represent the Pareto frontiers considered in reaction diagrams in Fig. 5.

XRD Results

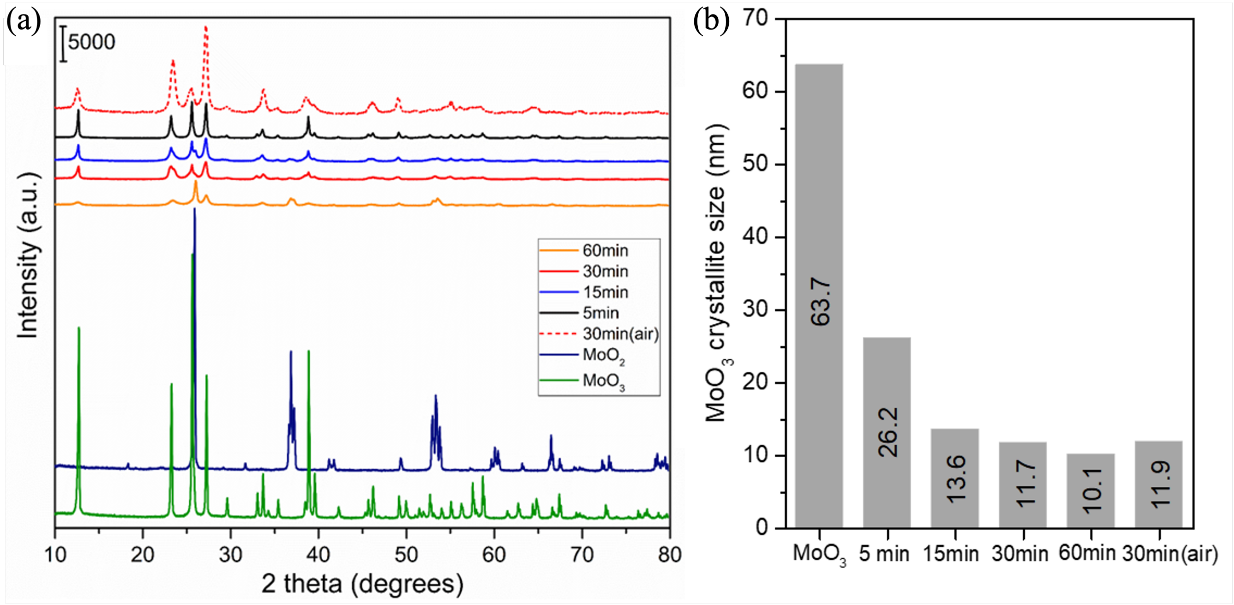

Figure S3: (a) X-ray diffraction patterns, and (b) MoO_3 crystallite sizes calculated by Rietveld refinement for the samples.

Table S1: Sample characteristics and crystallite sizes

Sample	% of the crystalline phases		MoO_3 crystallite sizes (nm)	Rwp*
	MoO_3	MoO_2		
MoO_3	100	0	64	11.656
60 min, 30 Hz	57.82	42.18	10	4.789
30 min, 30 Hz	77.08	22.92	12	8.695
15 min, 30 Hz	84.35	15.65	14	7.141
5 min, 30 Hz	96.59	3.41	26	7.375
30 min, 30 Hz (air)	92.70	7.30	12	6.790

*Weighted-profile R-factor for the refinement

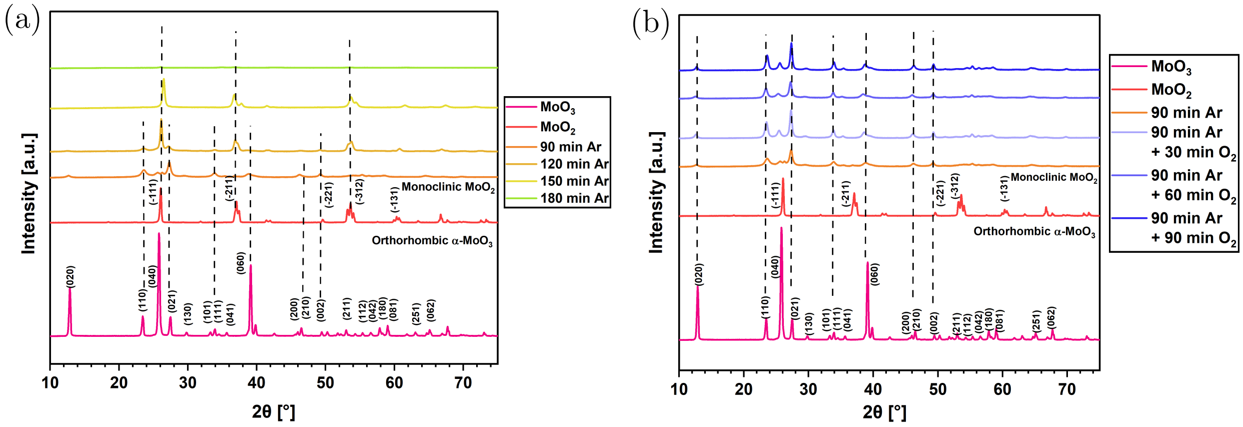

Figure S4: X-ray diffraction patterns of the MoO_3 samples milled in Ar for 90 min, followed by further milling in Ar (a) or O_2 (b) environments.

The XRD data indicates that the material transitions from orthorhombic α-MoO_3 to monoclinic MoO_2, with a gradual decrease in peak intensity over time, suggesting both amorphization and a shift to a different crystal structure. However, when the milling atmosphere was switched to O_2 after 90 minutes, the material retained its structure, maintaining the intensity observed at that time without further transition to MoO_2.

XANES Results

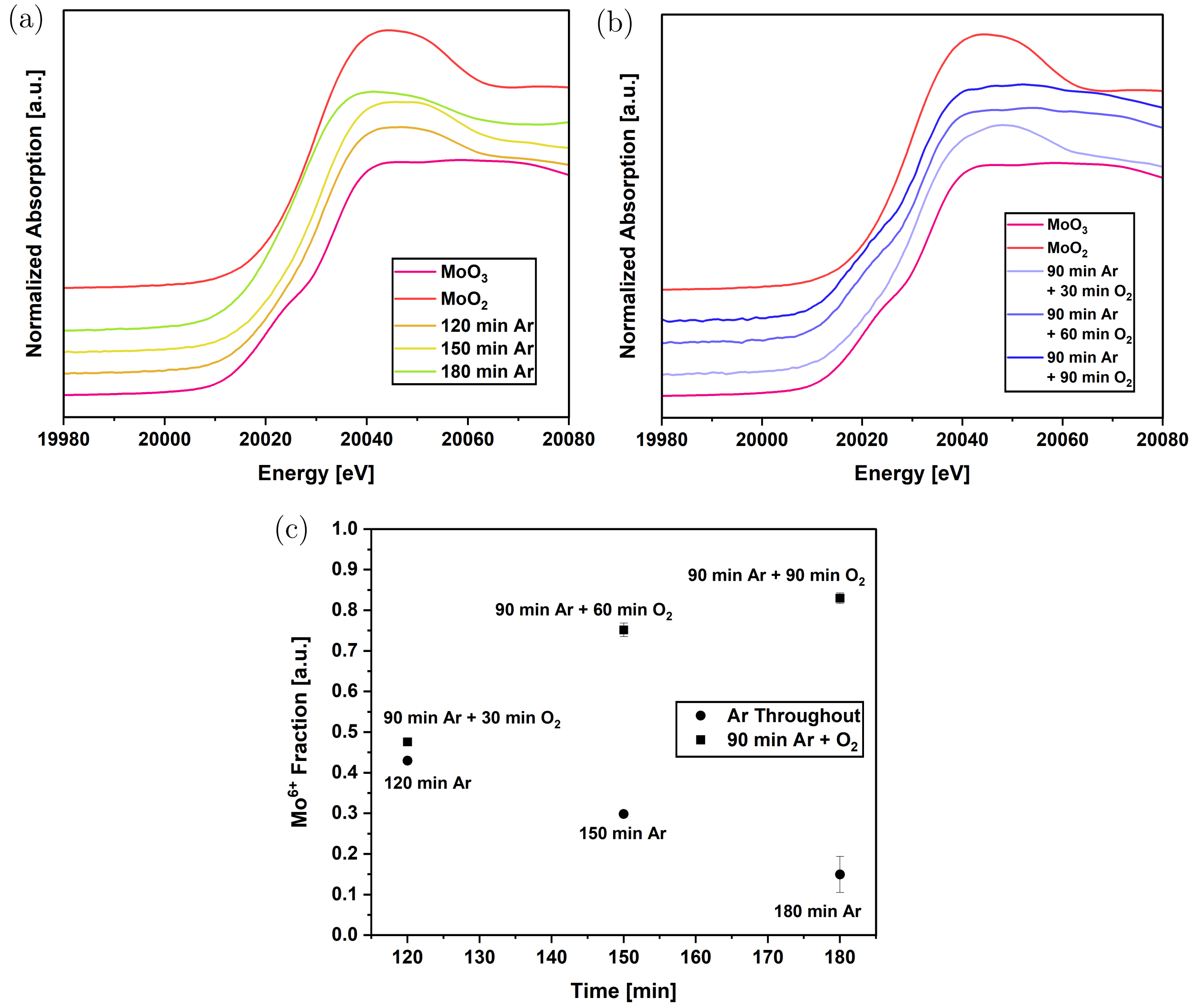

Figure S5: Mo K-edge XANES spectra for the standards (MoO_3 and MoO_2) and samples milled in Ar (a) or O_2 (b) environments. (c) Quantification of Mo(6+) in samples subjected to different milling times and environments at 30 Hz using a linear combination of XANES spectra.

Raman Spectra

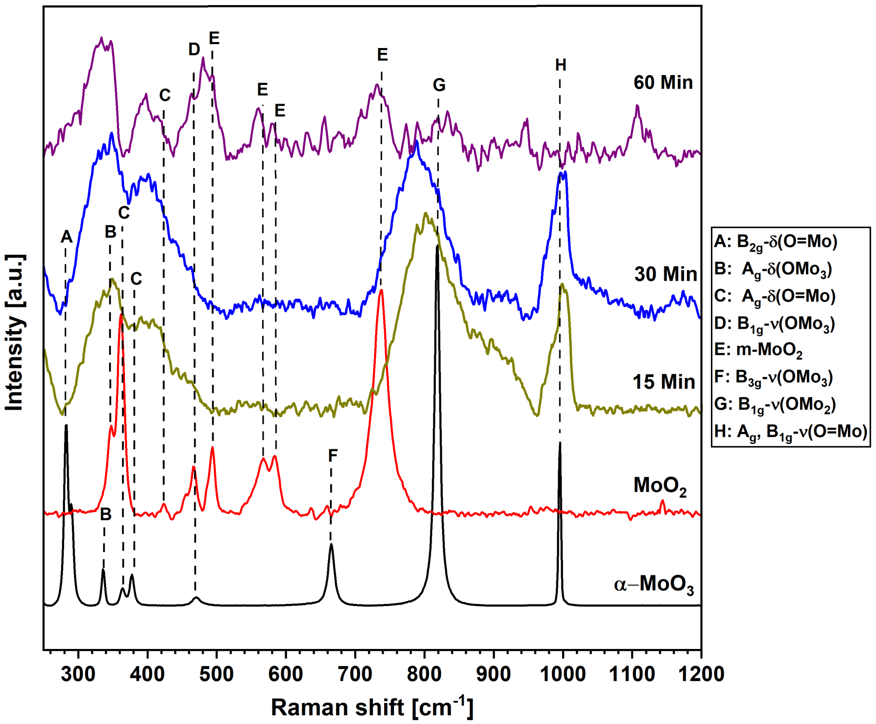

Figure S6: Raman spectra of the references (MoO_2 and MoO_3) and the MoO_3 milled for different times.[2]

All Raman spectra were gathered using the Renishaw inVia™ confocal Raman microscope equipped with a temperature-controlled CCD camera. Raman spectra were acquired using a 785 nm argon ion laser at 100x magnification of the objective. The laser penetration depth and spot area are 12 µm and 1.8 µm^2, respectively. The laser power at the sample was set to 0.1 % of the total power (150 mW) with 8 seconds of exposure time. In order to avoid structural changes in analyzed materials, including laser-induced oxidation of Mo($\delta+$) surface species, all spectra were obtained using a such low laser power. The consequence of reduced laser power is relatively low signal to noise ratio. The baseline correction was applied to each reported spectra using the Windows®-based Raman Environment (WiRE) software.

The collected Raman spectra reveal a clear transition from MoO_3 to MoO_2 with increasing milling time. The broadening of the bands is indicative of amorphization along with

significant particle size reduction (confirmed also by DLS) caused by the milling process. Specifically, bands associated with surface Mo=O bonds, located above 800 cm^{-1} for MoO_3, exhibit a decrease in intensity, while those around 700 cm^{-1}, corresponding to MoO_2, show an increase in intensity.[2] This indicates significant surface restructuring of the material. In the lower wavenumber region, the bands represent vibrations of the bulk-phase -Mo_n-O-. Although the appearance of multiple bands and their broadening make interpretation more challenging, a noticeable shift is observed with prolonged milling. By 60 minutes, the characteristic vibrations of bulk-phase bonds -Mo_n-O- become apparent, suggesting that the lattice structure undergoes substantial alteration at this stage.

Particle Size Analysis by DLS

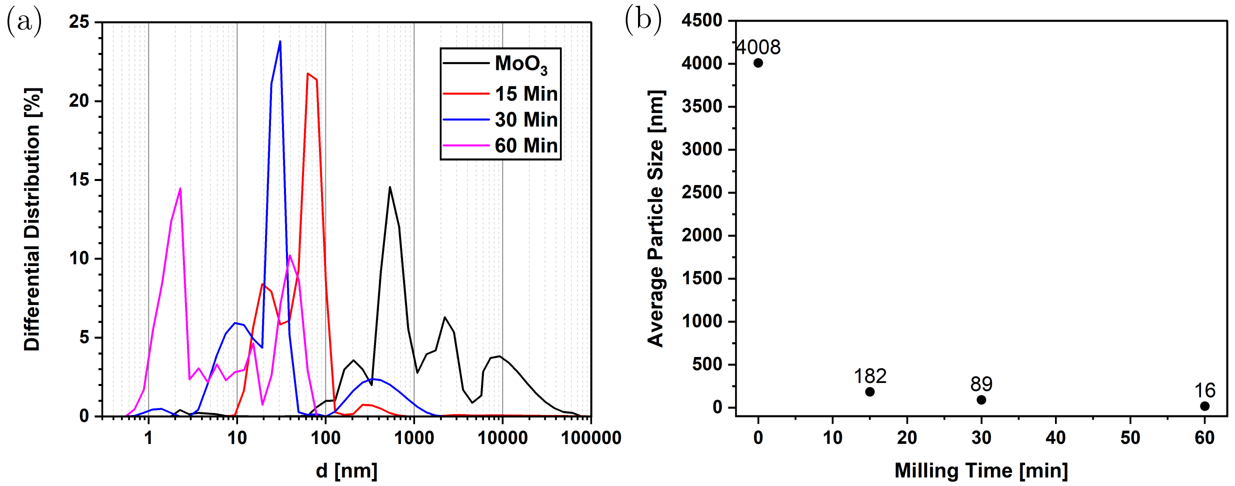

Figure S7: (a) Particle size distribution of the MoO_3 given by DLS measurements. (b) Average particle size calculated based on the particle size distribution.

The particle size distribution of all samples was measured using a Wyatt DynaPro NanoStar II dynamic light scattering instrument. Approximately 10 mg of the solid samples is dispersed in 1 mL of methanol in a 2 mL plastic centrifuge tube and transported into a plastic cuvette. For each sample, 10 measurements were taken with the acquisition time of 5 s. The particle size distribution as well the average particle size is calculated based on the average values of all measurements.

SEM Images

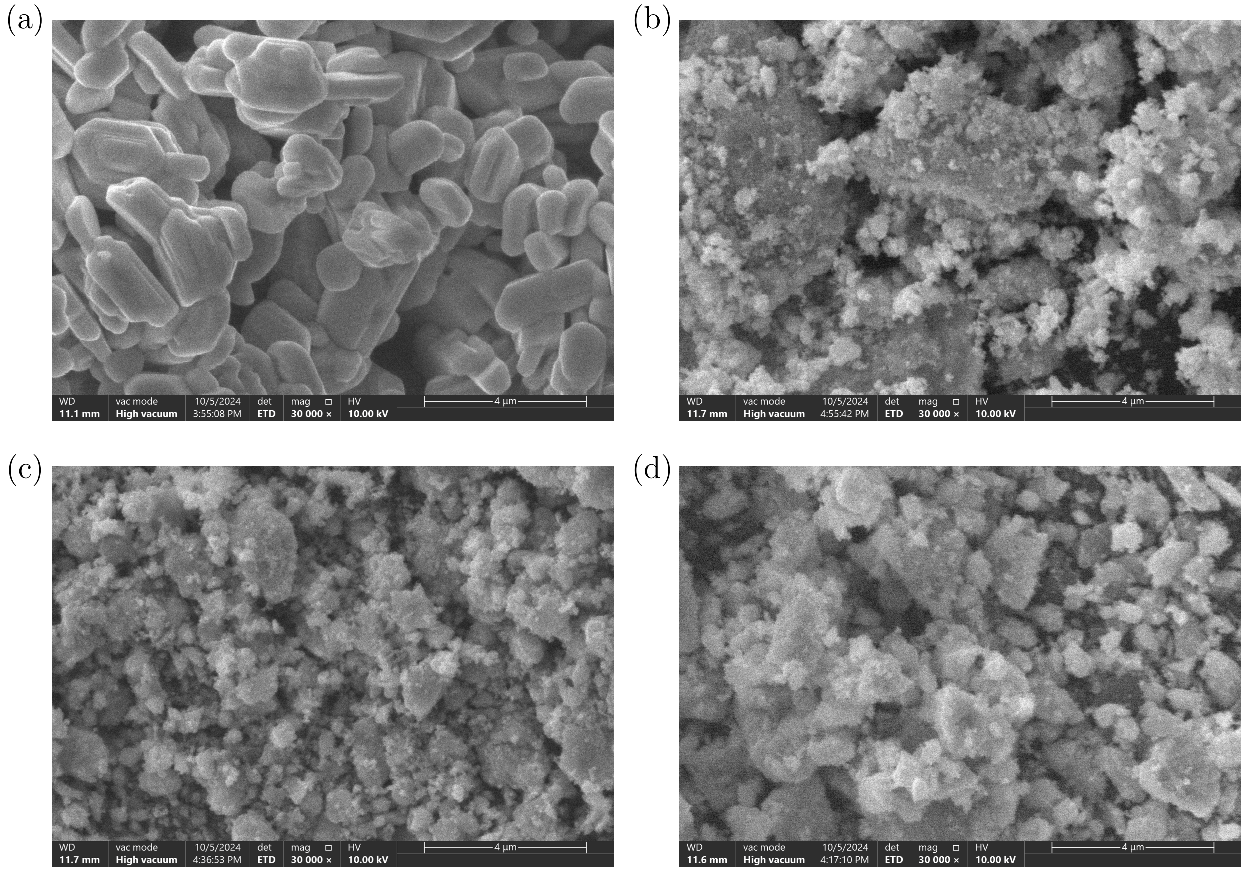

Figure S8: SEM images of the MoO_3 before milling (a) and after milling for 15 min (b), 30 min (c), and 60 min (d).

XPS Survey Scan

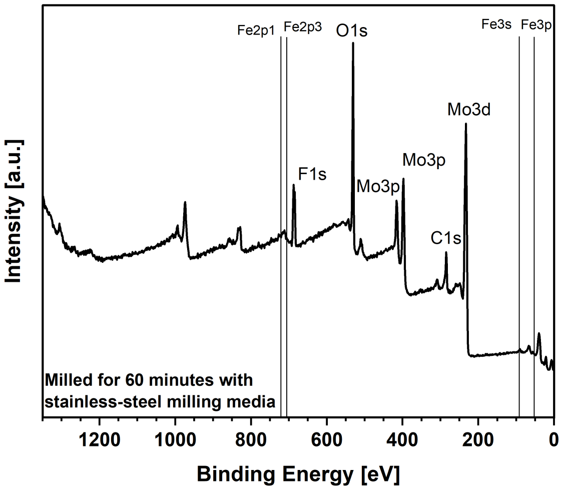

Figure S9: XPS survey scan of the MoO_3 sample milled for 60 min in stainless steel vessel with stainless ball

References

(1) Ressler, T.; Wienold, J.; Jentoft, R. E.; Neisius, T. Bulk structural investigation of the reduction of MoO3 with propene and the oxidation of MoO2 with oxygen. *Journal of Catalysis* **2002**, *210*, 67–83.

(2) Camacho-López, M. A.; Escobar-Alarcón, L.; Picquart, M.; Arroyo, R.; Córdoba, G.; Haro-Poniatowski, E. Micro-Raman study of the m-MoO2 to α-MoO3 transformation induced by cw-laser irradiation. *Optical Materials* **2011**, *33*, 480–484.

(3) Ressler, T.; Jentoft, R. E.; Wienold, J.; Günter, M. M.; Timpe, O. In situ XAS and XRD studies on the formation of Mo suboxides during reduction of MoO3. *Journal of Physical Chemistry B* **2000**, *104*, 6360–6370.

(4) Spevack, P. A.; McIntyre, N. S. Thermal reduction of molybdenum trioxide. *The Journal of Physical Chemistry* **1992**, *96*, 9029–9035.
